# Aspects cliniques et thérapeutiques du priapisme au CHU Gabriel Touré: étude de 36 cas

**DOI:** 10.11604/pamj.2014.17.286.4109

**Published:** 2014-04-15

**Authors:** Amadou Kassogué, Mamadou Coulibaly, Zanafon Ouattara, Alkadri Diarra, Aly Tembely, Mohamed Jamal El Fassi, Moulay Hassan Farih, Kalilou Ouattara

**Affiliations:** 1 Département de Chirurgie, Service d'Urologie, CHU Hassan II, Fès, Maroc; 2 Département de Chirurgie, Service d'Urologie, CHU Gabriel Touré, Bamako, Mali; 3 Département de Chirurgie, Service d'Urologie, CHU Point G, Bamako, Mali

**Keywords:** Priapisme, fonction érectile, drépanocytose, ponction des corps caverneux, priapism, erectile function, Sickle cell disease, puncture of the corpora cavernosa

## Abstract

Le priapisme est une érection prolongée douloureuse et irréductible survenant en dehors de toute stimulation sexuelle et n'aboutissant pas à une éjaculation. C'est une urgence urologique. Au Mali, la drépanocytose, une pathologie endémique joue un rôle de premier plan parmi les étiologies. L'objectif de cette étude est d'analyser les aspects cliniques et thérapeutiques du priapisme. Nous avons réalisé une étude rétrospective de type descriptive portant sur 36 cas de priapisme colligés au service d'urologie du CHU Gabriel Touré sur une période de 6 ans et 4 mois. L’âge moyen de nos patients était de 17 ans. Les tranches d’âge les plus représentées étaient comprises entre (11-20 ans) et (21-30 ans) soit 58%. 11 patients soit 31 % avaient eu un antécédent d’érection prolongée; le délai de consultation à partir du premier signe du priapisme était retardé, 31 % des patients étaient venus dans les 24-72h. Sur les 34 patients qui avaient fait l’électrophorèse de l'hémoglobine, 31 patients soit 91 % étaient porteurs d'hémoglobine anormale S et ou C. 32 patients soit 89% de nos patients avaient eu une ponction des corps caverneux. La détumescence a été obtenue le même jour chez 61 % des patients. Chez 11 patients soit 31 %, l’érection était bonne. Le priapisme est une urgence urologique, dont la fréquence est élevée dans la population drépanocytaire. La drépanocytose était la principale cause dans notre pays, tout praticien doit systématiquement y penser avant toute autre étiologie.

## Introduction

Le priapisme est une érection prolongée douloureuse et irréductible survenant en dehors de toute stimulation sexuelle et n'aboutissant pas à une éjaculation. C'est une urgence urologique. Le pronostic du priapisme traité est essentiellement fonction de sa durée et de l’étiologie, le vrai test de guérison n'est pas sa disparition seule mais aussi la récupération d'une fonction sexuelle normale. Plus le priapisme se prolonge, plus les chances de guérison diminuent, car s'installent des lésions irréversibles des corps érectiles responsables de cette dysfonction érectile [1]. Le priapisme est une pathologie très rare. Au Mali, la drépanocytose, une pathologie endémique joue un rôle de premier plan parmi les étiologies. La drépanocytose est une hémoglobinopathie héréditaire de transmission autosomique récessive. Les patients atteints par cette anémie sont particulièrement exposés au risque de priapisme. Au moins 40% des patients drépanocytaires rapportent des épisodes de priapisme. Les priapismes des drépanocytaires ont la particularité de débuter dans l'enfance et de menacer rapidement le pronostic érectile [2]. L'objectif de cette étude a été d'analysée les aspects cliniques et thérapeutiques du priapisme dans le service d'urologie du CHU Gabriel Touré de Bamako.

## Méthodes

Il s'agit d'une étude rétrospective de type descriptive portant sur 36 cas de priapisme colligés durant une période de 6 ans et 4 mois (1er mai 2005 au 31 octobre 2012). Ainsi, ont été inclues de notre étude tout malade reçu en consultation d'urgence ou ordinaire pour érection douloureuse, irréductible de plus de quatre heures et ayant réalisé le bilan sanguin au cours de l’épisode de priapisme. Ont été exclues de notre étude tout patient ayant une érection de moins de quatre heures ou plus de quatre heures reçues pendant la période d’étude, non enregistrées dans le registre de consultation d'urgence, ordinaire et du bloc opératoire.

La ponction ou l'incision des corps caverneux était systématique chez tous les patients ayant une érection de plus de douze heures en accord avec les patients (problème médicolégal). En cas de désaccord avec le patient pour la ponction, un traitement médical était instauré à base d'anti- inflammatoire, antalgique, anti-oedémateux, vasodilatateurs. Une consultation hématologique était donnée à chaque patient après le traitement pour un suivi de la drépanocytose. La ponction était effectuée soit au niveau du sillon balanopréputial (entre le gland et le corps caverneux), pour éviter une sclérose cicatricielle ; soit directement dans le corps caverneux. Cette ponction avait non seulement pour but de soulager les malades mais aussi de préciser le type physiopathologique du priapisme (de stase ou de haut débit). Les variables étudiées étaient d'ordre épidémiologique (l’âge du patient, le statut matrimonial), clinique (le délai de consultation, les antécédents d’épisode d’érection prolongé, le statut du génotype hémoglobinique) et thérapeutique (le temps de détumescence, le résultat du traitement).

## Résultats

**Figure 1:**
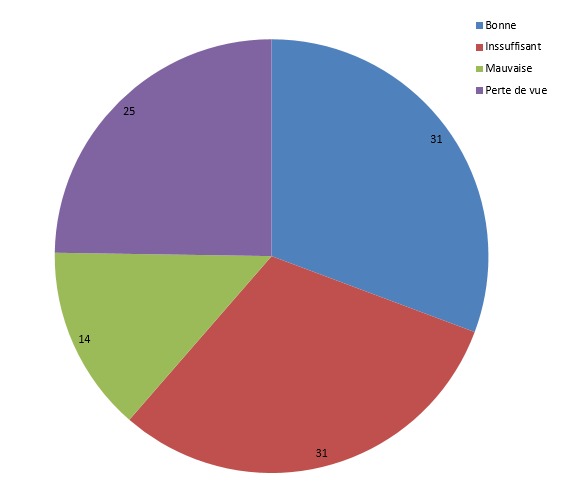
Répartition des patients selon la qualité d’érection post priapique

Durant cette période d’étude nous avions colligé 36 cas de priapisme. L’âge moyen de nos patients était de 17 ans avec des extrêmes allant de 5 à 60 ans. Les tranches d’âge les plus représentées étaient comprises entre (11-20 ans) et (21-30 ans) soit 58%. 23 patients soit 64% de nos patients étaient célibataires. 11 patients soit 31 % avaient eu un antécédent d’érection prolongée; le délai de consultation à partir du premier signe du priapisme était retardé, 31 % des patients étaient venus dans les 24-72h, 22% dans les 12-24h et 14% dans les 72h à 5 jours (Tableau 1). Sur les 34 patients qui avaient fait l’électrophorèse de l'hémoglobine 31 patients soit 91 % étaient porteurs d'hémoglobine anormale S et ou C, 16 % étaient drépanocytaires homozygotes SS, 8 % étaient drépanocytaires hétérozygotes AS, 7 % étaient drépanocytaires doubles hétérozygotes SC. Deux patients étaient porteurs d'hémoglobine normale AA (Tableau 2). 32 patients soit 89% de nos patients avaient eu une ponction des corps ca verneux. La détumescence a été obtenue le même jour chez 61 % des patients ponctionnés. Chez les autres chez 25% la détumescence n'a été complète qu'entre le 2eme et le 5eme jour après la ponction et 14% entre le 6ème et 10ème (Tableau 3). Chez 11 patients soit 31 % l’érection était bonne. Chez 31 % des patients l’érection étaient insuffisante et chez 14 % des patients l’érection était absente après le traitement (Figure 1).

**Tableau 1:** Répartition des patients selon le délai de consultation à partir du premier signe du priapisme

Délai de consultation	Effectif	%
4h-6h	4	11
6h-12h	4	11
12h-24h	8	22
24h-72h	11	31
72h-5j	5	14
>1 semaine	4	11
Total	36	100

Le retard de consultation a été énorme, 78% des patients sont venu après les 12 heures

**Tableau 2:** Répartition des patients selon le type d'hémoglobine

Type d'hémoglobine	Effectif	%
SS	16	44
AS	8	22
SC	7	19
AA	3	8
Indéterminé	2	6
Total	36	100

Plusieurs cas de priapisme sont survenus sur terrain drépanocytaire, soit 86 % des cas et la forme SS a représenté 44%

**Tableau 3:** Répartition des patients selon le temps de détumescence en jour après le traitement médical et la ponction

Temps de tumescence en jour après le traitement	Effectif	%
0-1	22	61
2-5	9	25
06-10	5	14
Total	36	100

Chez 61% des patients, la détumescence a été obtenue dès le premier jour

## Discussion

En 6 ans et 4 mois nous avions colligé 36 cas de priapisme dont 32 cas survenu sur terrain drépanocytose. Entre 1845 et 1914, seulement 172 cas de priapisme ont été rapportés dans la littérature soit environ 2 cas par an dans le monde [3]. Notre taux est élevé par rapport à celui de l’étude de Benchekroun. A qui avait recensé 16 cas en 15 ans soit environ un cas par an [4]. Nettement inférieur à celui de R. Virag qui avait eu 172 cas en 10 ans, soit 17 cas de priapisme environ par an [3]. L’âge moyen de notre étude est de 17 ans, ce qui est légèrement supérieur à celui de la littérature ou l’âge moyen dans les zones d'endémies drépanocytaires est situé à moins de 15 ans. Les tranches d’âge les plus représentées dans notre étaient comprises entre (11-20 ans) et (21-30 ans) soit 58% qui est similaire à l’étude de Latoundji et al. [5] avec un pic de fréquence dans la tranche d’âge de 21 à 30. Cette moyenne d’âge est similaire est inferieur à l’étude de P.A. Bouya et al. qui ont eu 23 ans comme moyen d’âge [6]. Contrairement à beaucoup études ou la drépanocytose occupe une place moindre dans l’étiologie, elle est de 91 % dans notre étude. R. Virag [3] avait reçu 3,4 % de drépanocytose contre 86,6 % de complications des IIC. Benchekroun. A [4] avait trouvé un drépanocytaire sur 16 patients soit 6 % des malades. Certaine étude [6] avait eu 34 % d’étiologie drépanocytaire et 34 % iatrogène. Celle de Latoundji S et al. avait trouvé que la drépanocytose est la principale cause de priapisme avec 88,89 % et que les homozygotes SS (75 %) sont plus exposés que les doubles hétérozygotes SC(16,67 %) et les hétérozygotes AS (8,33 %). Ils constataient également que le priapisme drépanocytaire était plus fréquent chez l'adulte jeune de 21 à 35 ans (66,67 %) que chez l'enfant de 5 à 15 ans (20,83 %) ; et que le maximum de fréquence se situait entre 20 et 30 ans (50 %) ce qui est similaire à notre étude.

Il s´agit d´une urgence médicochirurgicale dont la découverte chez le noir impose la recherche de la drépanocytose par la réalisation de l´électrophorèse de l´hémoglobine. La survenue d´épisodes aigus succédant au priapisme intermittent ou chronique avec altération de la fonction érectile est la hantise au cours de ce phénomène [7]. La récupération d'une érection normale dépend de trois facteurs pronostics majeurs chez le drépanocytaire : le délai écoulé entre le début du priapisme et la mise en route du traitement, l’âge du patient et le nombre de crise antérieure. Le priapisme veineux est beaucoup plus péjoratif, notamment chez les drépanocytaires car ils sont exposés à de nombreuses récidives. La fonction érectile se dégrade progressivement et cède parfois brutalement au décours d'une crise vaso-occlusive vers une impuissance définitive. Le pronostic du priapisme artériel paraît meilleur puisque certains patients, dont le priapisme évoluait depuis plusieurs mois, ont retrouvé une puissance sexuelle normale [2]. Une prise en charge chirurgicale précoce permet d'augmenter les chances de conservations de la fonction érectile. Ce n'est pas la chirurgie qui altère la fonction érectile mais la durée du priapisme. Les facteurs pronostics péjoratifs responsables d'une altération de la fonction érectile identifiées dans leurs études étaient l'augmentation de la durée et la répétition des épisodes priapiques [8]. Vu le délai écoulé entre l'apparition des premiers signes du priapisme et le début de sa prise en charge, notre taux de dysfonctionnement sexuel pourrait s'expliquer par le fait de ce retard.

## Conclusion

Le priapisme est une urgence urologique, aux complications redoutables, dont la fréquence est considérablement élevée dans la population drépanocytaire. La drépanocytose a été la principale cause dans notre pays, tout praticien doit systématiquement y penser avant toute autre étiologie. La prise en charge précoce permet d'améliorer la qualité du dysfonctionnement érectile.
